# ﻿Description of five new *Luticola* D.G.Mann (Bacillariophyta, Diadesmidaceae) species from Indonesia with comments on the morphological boundaries of the genus

**DOI:** 10.3897/phytokeys.237.113773

**Published:** 2024-01-05

**Authors:** Mateusz Rybak, Łukasz Peszek, Oktiyas Muzaky Luthfi, Sulastri Arsad, John Patrick Kociolek, Andrzej Witkowski

**Affiliations:** 1 Department of Agroecology and Forest Utilization, University of Rzeszów, ul. Ćwiklinskiej 1A, 35-601 Rzeszów, Poland University of Rzeszów Rzeszów Poland; 2 University of Brawijaya, Department of Marine Science, Malang, East Java, Indonesia University of Brawijaya Malang Indonesia; 3 Museum of Natural History and Department of Ecology and Evolutionary Biology, University of Colorado, Boulder, CO, USA University of Szczecin Szczecin Poland; 4 University of Szczecin, Institute of Marine and Environmental Sciences, Mickiewicza 16A, 70-383 Szczecin, Poland University of Colorado Boulder United States of America

**Keywords:** Diatoms, morphology, Southeast Asia, taxonomy

## Abstract

During a survey of the Indonesian diatoms, five *Luticola* D.G.Mann taxa that could not be identified, based on the available literature were discovered. Based on light microscopy, scanning electron microscope observations and comparisons with similar taxa, all of them are described as new species. All taxa were found on mosses growing on tree trunks and concrete on the islands of Banda Besar and Seram and from spring on Java Island. *Luticolainsularis***sp. nov.** is most similar to *L.aequatorialis* and *L.simplex*, but it can easily be distinguished from both taxa, based on the lower striae density, the narrower valves and the well-developed silica ridges on the valve face/mantle junction. *Luticolabandanensis***sp. nov.** resembles *L.frequentissima*, but they can be easily distinguished, based on their valve widths and the direction of the grooves located on the distal and proximal raphe endings. *Luticolaelliptica***sp. nov.** is most similar to *L.sparsipunctata*, *L.tenuis* and *L.bryophila*. Amongst all the species compared, *L.elliptica***sp. nov.** is the only one with a highly asymmetrical central area, with the isolated pore located on the wider side. *Luticolamalukuana***sp. nov.** shares similarities with *L.dismutica* and *L.areolata*, but it has a notably higher stria density. From *L.areolata*, it can also be separated by the morphology of striae and the lack of ghost areolae in the central area. *Luticolapoliporea***sp. nov.** is unique in the whole genus due to the presence of multiple isolated pores.

## ﻿Introduction

The study of terrestrial diatoms in Southeast Asia began at the end of the 19^th^ century (Grunow 1865) and continues to this day. The result of this work is over 100 scientific publications focusing on diatoms in this area (Glushchenko et al. 2021). Despite a relatively long history of diatom research, terrestrial and aerophytic diatoms have received much less attention. The first and, for a long time, the only mention of this ecological group in Southeast Asia was the work of Kolkwitz and Krieger (1936), in which they mention the results from a single soil sample collected under trees, in which they found just a few diatom species. However, the situation has been changing in recent years, reflected in the descriptions of many new species from terrestrial environments of this region like soils or tufts of mosses (Kezlya et al. 2020a, b, 2022a, b; Rybak et al. 2020, 2022a, b, c; Glushchenko et al. 2022).

Many genera of diatoms are recorded in terrestrial and aerophytic environments and *Luticola* D.G. Mann (in Round et al. (1990: 148)) seems to be one of the most diverse (Chattová 2018; Bishop et al. 2021; Rybak et al. 2021a, 2023; Radhakrishnan et al. 2022; Chattová et al. 2022). However, representatives of this genus can also be found in other environments such as fresh-, brackish and marine water and even as epizoic on turtles (Wetzel et al. 2010; Levkov et al. 2013; Wu and Bergey 2017; Rybak et al. 2021b). The genus *Luticola* was distinguished from *Navicula* to accommodate species included in the Naviculaesect.Punctatae with Luticolamutica (Kützing) D.G.Mann (in Round et al. (1990: 532)) selected as the generitype. Features common to all species are the distinctly punctate, uniseriate striae composed of rounded to elongated areolae covered internally by perforated hymenes, an evident and morphologically unique isolated pore in the central area and a marginal longitudinal channel positioned internally between the valve face and the valve mantle (Round et al. 1990; Levkov et al. 2013). Since the publication of the Levkov’s et al. (2013) monograph on the genus, in which almost 200 species are treated, the genus has attracted the attention of many taxonomists from around the world. As a result of their work, the number of currently-known taxa has increased to 262 (Guiry and Guiry 2023), amongst which 23 species have been described from tropical Asia (Glushchenko et al. 2017; Liu et al. 2017; Lokhande et al. 2020; Rybak et al. 2021b; Yang et al. 2022) or transferred to the genus (Glushchenko and Kulikovskiy 2015; Kale et al. 2017).

In this paper, five new *Luticola* taxa are described from terrestrial and water mosses in Indonesia and separated from other similar taxa, based on their combinations of morphological features as documented with light and scanning electron microscopy.

## ﻿Material and methods

The Maluku Islands (Spice Islands or Moluccas) are an archipelago in the northeast of Indonesia. The climate of the study area is almost entirely tropical and is dominated by a tropical rainforest climate with wet and dry seasons. Samples of terrestrial mosses from concrete and tree trunks were collected, placed in paper envelopes and left to dry. Moss samples from springs were collected with a spoon and placed in a plastic container. Three samples, in which unidentified *Luticola* taxa were observed, were selected for this study:

- 2018/440 – 4°31'29.93"S, 129°56'51.69"E, terrestrial orthotropic mosses collected from the base of a tree trunk on Banda Besar, Indonesia, in a forest near the shore at an elevation of 19 m a.s.l. The pH measured was 5.8 and conductivity was 1250 µS/l/cm.
- 2018/447 – 3°19'12.2"S, 128°56'6.94"E, terrestrial plagiotropic mosses collected from concrete in Amahai, Seram, Indonesia, at an elevation of 14 m a.s.l. The pH measured was 6.7 and conductivity was 680 µS/l/cm.
- 2023/81 – 7°50'26.2"S, 112°31'43.0"E, plagiotropic mosses from an unnamed spring collected in Malang, East Java, Indonesia.

The samples were used for preparation of diatom slides and filtrates for pH and conductivity measurements. The filtrates were obtained by soaking pieces of moss in deionised water (at a 1:10 weight ratio) for 24 h. pH and electrical conductivity were measured with a MARTINI pH56 pH meter and a MARTINI EC59 conductivity meter (Szeged, Hungary).

For diatom slides preparation, a small part of each moss sample was digested with a mixture of sulphuric acid and potassium dichromate. After dissolving all organic matter, the suspension was centrifuged at 2500 rpm to remove the dissolving mixture and subsequently washed 3–5 times with centrifugation in distilled water. The cleaned diatom suspension was pipetted on to coverslips, left to dry overnight at room temperature and then mounted with Naphrax (Brunel Microscopes Ltd, Wiltshire, U.K.). Identification, counting and the measurements of the diatoms’ basic morphological features were performed under a Carl Zeiss Axio Imager A2 light microscope (LM), equipped with a 100× Plan Apochromatic objective with differential interference contrast (DIC) for oil immersion (NA 1.4) and captured with a Zeiss AxioCam ICc5 camera. For scanning electron microscope (SEM) observations, several drops of the samples were placed on a polycarbonate membrane filter with a 3 μm mesh, attached to aluminium stubs and sputtered with 20 nm of gold using a Turbo-Pumped Sputter Coater Quorum Q 150OT ES. The diatoms were observed using a Hitachi SU 8010 SEM. Diatom terminology follows Barber and Haworth (1981), Round et al. (1990) and Levkov et al. (2013).

## ﻿Results

### ﻿ Luticola insularis

Taxon classificationPlantaeNaviculalesDiadesmidaceae

M.Rybak & Peszek sp. nov.

0B582ED2-9B19-56F6-B44A-70D88CDC2E81

Figs 1A–S , 4A–H

#### Description.

**Figure 1.**
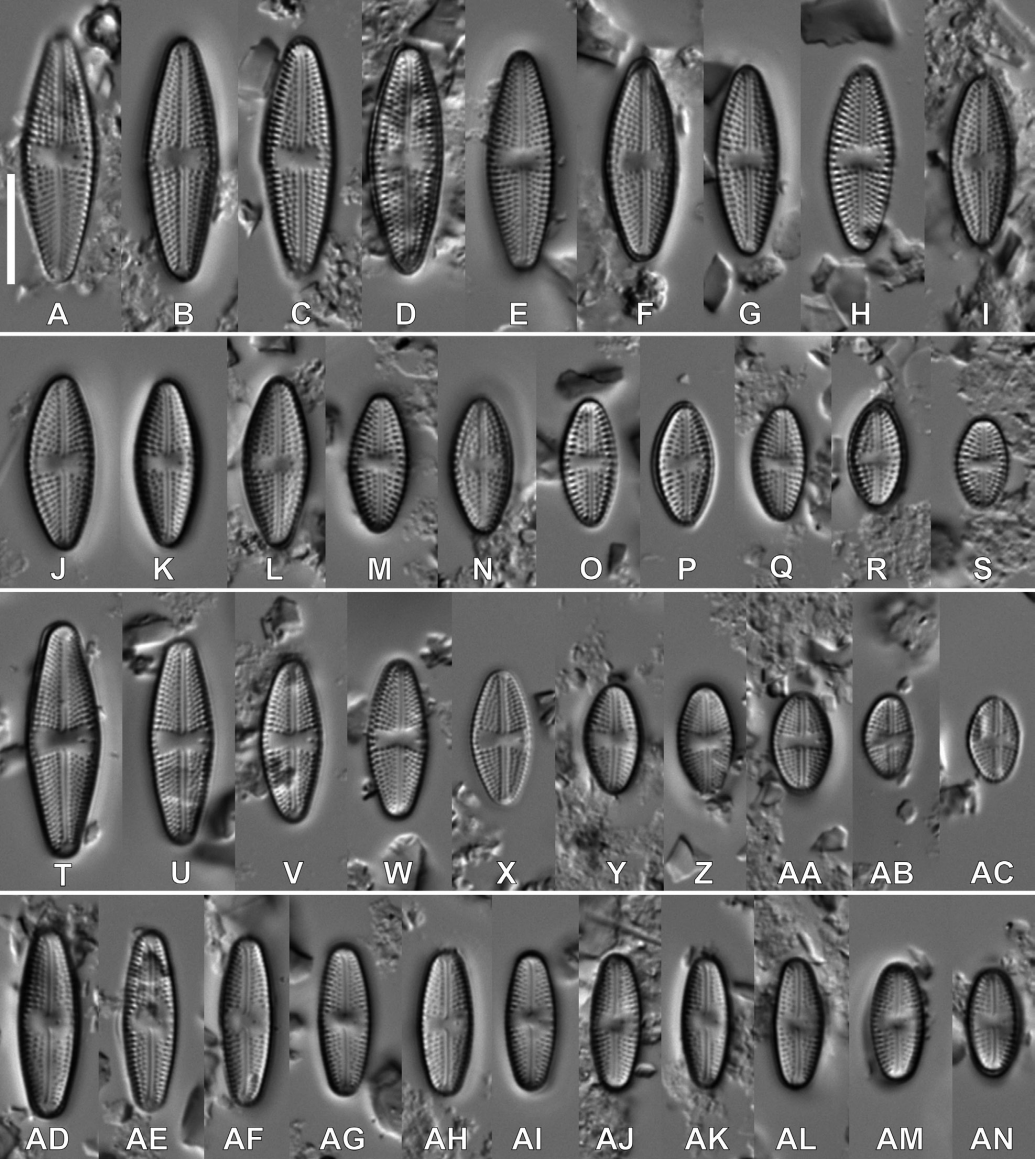
LM microphotographs of three new *Luticola* taxa in size diminution series. *Luticolainsularis* sp. nov. (**A–S**), *Luticolabandanensis* sp. nov. (**T–AC**), *Luticolaeliptica* sp. nov. (**AD–AN**). Scale bar: 10 µm.

**LM observations (Fig. 1A–S).** Valves elliptic-lanceolate to lanceolate with narrowly rounded apices. Range of valve dimensions (n = 35): 8.5–24.0 μm long, 4.5–7.0 μm wide and 16–20 striae in 10 µm. Striae clearly punctate, composed of 3–4 areolae. Central area rectangular to slightly bow-tie shaped axial area linear and narrow Isolated pore clearly visible in central area located halfway between the margin and the centre. Raphe filiform with proximal endings slightly bent away from isolated pore, distal raphe endings barely visible.

**SEM observations (Fig. 4A–H). *External view***: Striae composed of 3–4 areolae round, becoming larger and more elongated near the margins (Fig. 4A–E). Single row of elongated areolae, interrupted at valve apices, present on valve mantle (Fig. 4D, F). Central area bordered by 2–3 areolae (Fig. 4A–D). Raphe filiform with weakly hooked distal ends continuing on to valve mantle (Fig. 4A, C–E). Proximal raphe ends slightly bent opposite to the isolate pore bearing side, then shortly hooked to the pore and finishing with a small, elongated shallow depressions (Fig. 4B, D, E). Opening of isolated pore small and slightly elongated (Fig. 4A–D). Girdle bands open with two rows of poroids (Fig. 4D, F). Distinct silica ridge visible along valve face/mantle junction (Fig. 1D, F).

***Internal view***: Areolae occluded by hymenes forming a continuous strip (Fig. 4G, H). Raphe slits straight (Fig. 4G, H). Proximal raphe endings simple and straight (Fig. 4G, H), distal raphe endings finishing on to weakly developed helictoglossae (Fig. 4G, H). Isolated pore opening with small lipped structure (Fig. 4G, H). Longitudinal channels visible along valve (Fig. 4G, H).

#### Type.

Indonesia, Banda Besar, 4°31'29.93"S, 129°56'51.69"E, 19 m a.s.l., coll. 23 June 2018, holotype slide no. SZCZ29103! and the unmounted material with the same number in the Szczecin Diatom Collection (University of Szczecin, Poland), isotype slide no. 2018/440 and the unmounted material with the same number at the University of Rzeszów, Poland. The type population is illustrated in Figs 1A–S, 4A–H.

#### Etymology.

The name refers to the fact that the species was found on one of many Asian islands (lat. *insula* – island).

#### Distribution.

So far, this species has been observed only in the type locality.

#### Ecology and associated diatom flora.

The species was observed in a sample characterised by a slightly acidic pH (5.8) and conductivity was 1250 µS/cm. The species described herein co-occurred with: *Luticolabandanensis* sp. nov., *L.elliptica* sp. nov., *L.minima* Levkov, Metzeltin & Pavlov, two unidentified small-celled *Nitzschia* sp., *Nitzschiavitrea* G.Norman and *Tryblionelladebilis* Arnott ex O’Meara.

### ﻿ Luticola bandanensis

Taxon classificationPlantaeNaviculalesDiadesmidaceae

M.Rybak & Peszek sp. nov.

22E0AC5A-4974-5A66-A897-0CC5EB44401D

Figs 1T –AC, 5A–H

#### Description.

**LM observations (Fig. 1T–AC).** Valves elliptic-lanceolate to rhombic-lanceolate with narrowly rounded apices in larger specimens becoming elliptic with broadly rounded apices in smaller specimens. Range of valve dimensions (n = 25): 6.8–21.5 μm long, 4.5–6.5 μm wide and 21–23 striae in 10 µm. Striae clearly punctate composed of 3–5 areolae. Central area bow-tie shaped, axial area linear and narrow. Isolated pore clearly visible in central area, shifted slightly to valve margin. Raphe filiform with proximal endings slightly bent away from isolated pore, distal raphe endings barely visible.

**SEM observations (Fig. 5A–H). *External view***: Striae composed of 3–5 round to slightly elongated areolae (Fig. 5A–F). Single row of round areolae present on valve mantle uninterrupted at the apices (Fig. 5D, E). Central area bordered by 2–4 areolae (Fig. 5A–F). raphe straight and filiform with distal raphe endings hooked with irregular shallow grooves on valve face on isolated pore side (Fig. 5A–F). Proximal raphe endings deflected finishing with varying in length shallow grooves extending to the first or second row of areolae on the opposite side of the isolated pore (Fig. 5A–F). Distal raphe endings hooked with irregular shallow grooves on isolated pore side (Fig. 5A–F). Small, transacially elongated opening of isolated pore shifted slightly to valve margin (Fig. 5A–F). Girdle bands open with two rows of poroids (Fig. 5D).

***Internal view***: Areolae occluded by hymenes forming a continuous strip (Fig. 5H). Raphe slits straight (Fig. 5G, H). Proximal raphe endings simple and straight (Fig. 5G, H), distal raphe endings finishing on to weakly developed helictoglossae (Fig. 5G, H). Isolated pore opening with small lipped structure (Fig. 5G, H). Narrow longitudinal channels visible around valve (Fig. 5G, H).

#### Type.

Indonesia, Banda Besar, 4°31'29.93"S, 129°56'51.69"E, 19 m a.s.l., coll. 23 June 2018, holotype slide no. SZCZ29103! and the unmounted material with the same number in the Szczecin Diatom Collection (University of Szczecin, Poland), isotype slide no. 2018/440 and the unmounted material with the same number at the University of Rzeszów, Poland. The type population is illustrated in Figs 1T–AC, 5A–H.

#### Etymology.

Name refers to type locality, the island of Banda Besar in the Banda Island archipelago in the Banda Sea.

#### Distribution.

So far, species observed only in the type locality.

#### Ecology and associated diatom flora.

The species was observed in a sample characterised by a slightly acidic pH (5.8) and conductivity was 1250 µS/cm. The species co-occurred with: *Luticolainsularis* sp. nov., *L.elliptica* sp. nov., *L.minima* Levkov, Metzeltin & Pavlov, two unidentified small-celled *Nitzschia* sp., *Nitzschiavitrea* G.Norman and *Tryblionelladebilis* Arnott ex O’Meara.

### ﻿ Luticola elliptica

Taxon classificationPlantaeNaviculalesDiadesmidaceae

M.Rybak & Peszek sp. nov.

4D41DAB7-9FBA-5BB0-B013-08AE87322A1C

#### Description.

**LM observations (Fig. 1AD–AN).** Valves elliptic with broadly rounded apices. Range of valve dimensions (n = 20): 9.0–20.0 μm long, 4.3–5.5 μm wide and 20–24 striae in 10 µm. Striae clearly punctate. Central area asymmetrical, side with isolated pore wider than the opposite site, axial area linear and narrow becoming slightly wider near to central area. Isolated pore clearly visible in central area located halfway between the margin and the centre. Raphe filiform with proximal endings slightly bent away from isolated pore, hooked distal raphe endings visible.

**SEM observations (Fig. 6A–H). *External view***: striae composed of 2–3 round to slightly elongated areolae (Fig. 6A–G). Single row of round areolae, interrupted at valve apices, present on valve mantle (Fig. 6A–D, F). Central area bordered by 4–7 areolae on side with isolated pore and 2–4 on opposite side (Fig. 6A–G). Raphe filiform with weakly-hooked distal endings continuing on to valve mantle (Fig. 6A–G). Proximal raphe endings deflected away from isolated pore and slightly enlarged (Fig. 6A–G). Opening of isolated pore round and not connected by areolae with striae (Fig. 6A–G). Girdle bands open with two rows of poroids.

***Internal view***: Areolae occluded by hymenes forming a continuous strip (Fig. 6H). Raphe slits straight (Fig. 6H). Proximal raphe endings simple and straight (Fig. 6H), distal raphe endings forming finishing on to weakly-developed helictoglossae (Fig. 6H). Isolated pore opening with small lipped structure (Fig. 6H). Longitudinal channels visible along valve (Fig. 6H).

#### Type.

Indonesia, Banda Besar, 4°31'29.93"S, 129°56'51.69"E, 19 m a.s.l., coll. 23 June 2018, holotype slide no. SZCZ29103! and the unmounted material with the same number in the Szczecin Diatom Collection (University of Szczecin, Poland), isotype slide no. 2018/440 and the unmounted material with the same number at the University of Rzeszów, Poland. The type population is illustrated in Figs 1AD–AN, 6A–H.

#### Etymology.

Name refers to the valve shape of the species.

#### Distribution.

Species has been observed in various locations in Southeast Asia. It was also observed on Borneo Island, Palambak Island, Sulawesi Island and Banda Besar (Rybak – unpublished data).

#### Ecology and associated diatom flora.

The species was observed in a sample characterised by a slightly acidic pH (5.8) and conductivity was 1250 µS/cm. The species co-occurred with: *Luticolainsularis* sp. nov., *L.bandanensis* sp. nov., *L.minima* Levkov, Metzeltin & Pavlov, two unidentified small-celled *Nitzschia* sp., *Nitzschiavitrea* G.Norman and *Tryblionelladebilis* Arnott ex O’Meara.

### ﻿ Luticola malukuana

Taxon classificationPlantaeNaviculalesDiadesmidaceae

M.Rybak & J.P. Kociolek sp. nov.

7E6901DF-DFDB-5D74-BC90-5D84F96EC79F

Figs 2A–S , 7A–H

#### Description.

**Figure 2.**
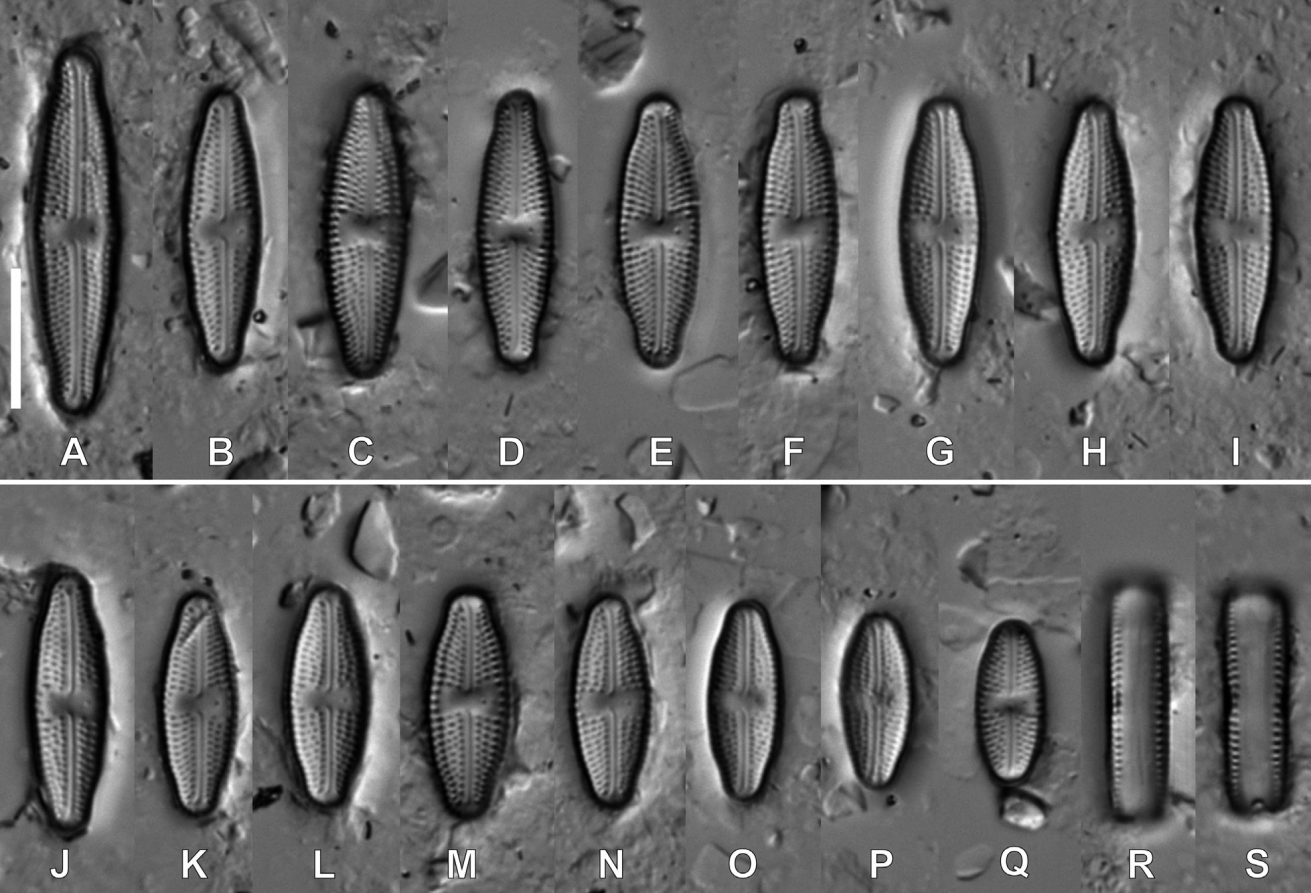
LM microphotographs of *Luticolamalukuana* sp. nov. in size diminution series. W, S – frustule in girdle view. Scale bar: 10 µm.

**LM observations (Fig. 2A–S).** Frustules rectangular in girdle view, larger valves linear-lanceolate with weakly-protracted apices and slightly undulating margin, smaller valves elliptic-lanceolate. Range of valve dimensions (n = 35): 12.0–28.0 μm long, 5.5–7.0 μm wide and 18–21 striae in 10 µm. Striae clearly punctate. Central area rectangular to slightly bow-tie-shaped, axial area linear and narrow. Isolated pore clearly visible in central area. Raphe filiform with proximal endings slightly bent away from isolated pore, distal raphe endings barely visible.

**SEM observations (Fig. 7A–H). *External view***: Striae composed of 3–4 round to slightly elongated areolae (Fig. 7A–D). Single row of elongated areolae, interrupted at valve apices, present on valve mantle (Fig. 7B, D). Central area bordered by 3–4 areolae (Fig. 7A–D). Raphe filiform with weakly-hooked distal endings continuing on to valve mantle (Fig. 7A–D). Proximal raphe endings deflected away from isolated pore (Fig. 7A–D). External opening of isolated pore round and not associated with striae (Fig. 7A–D). Girdle bands numerous and open with two rows of poroids (Fig. 7E, F).

***Internal view***: Areolae occluded by hymenes forming continuous strips (Fig. 7G, H). Raphe slits straight (Fig. 7G, H). Proximal raphe endings simple and straight (Fig. 7G, H). Isolated pore opening with small circular structure (Fig. 7G, H). Longitudinal channels visible along valve (Fig. 7G).

#### Type.

Indonesia, Seram, Amahai City, 3°19'12.2"S, 128°56'6.94"E, 14 m a.s.l., coll. 29 June 2018, holotype slide no. SZCZ29104! Additionally, the unmounted material with the same number in the Szczecin Diatom Collection (University of Szczecin, Poland), isotype slide no. 2018/447 and the unmounted material with the same number at the University of Rzeszów, Poland. The type population is illustrated on Figs 2A–S, 7A–H.

#### Etymology.

Name refers to Maluku Islands where the species was found.

#### Distribution.

So far, this species has been observed only in the type locality.

#### Ecology and associated diatom flora.

The species was observed in a sample characteried by a circum-neutral pH (6.7) and conductivity of 680 µS/cm. The species was the most abundant taxon in an assemblage including: *Humidophilalacunosa* (Moser, Lange-Bertalot & Metzeltin) Lowe, Kociolek, Johansen, Van de Vijver, Lange-Bertalot and Kopalová, *Luticolahustedtii* Levkov, Metzeltin & Pavlov, *Luticolaintermedia* Levkov, Metzeltin & Pavlov and *Luticolasublagerheimii* Levkov, Metzeltin & Pavlov.

### ﻿ Luticola poliporea

Taxon classificationPlantaeNaviculalesDiadesmidaceae

M.Rybak, Peszek, Luthfi, Arsad & A.Witkowski sp. nov.

FED92250-AE36-5391-B544-D27A603B7B68

**Figure 3.**
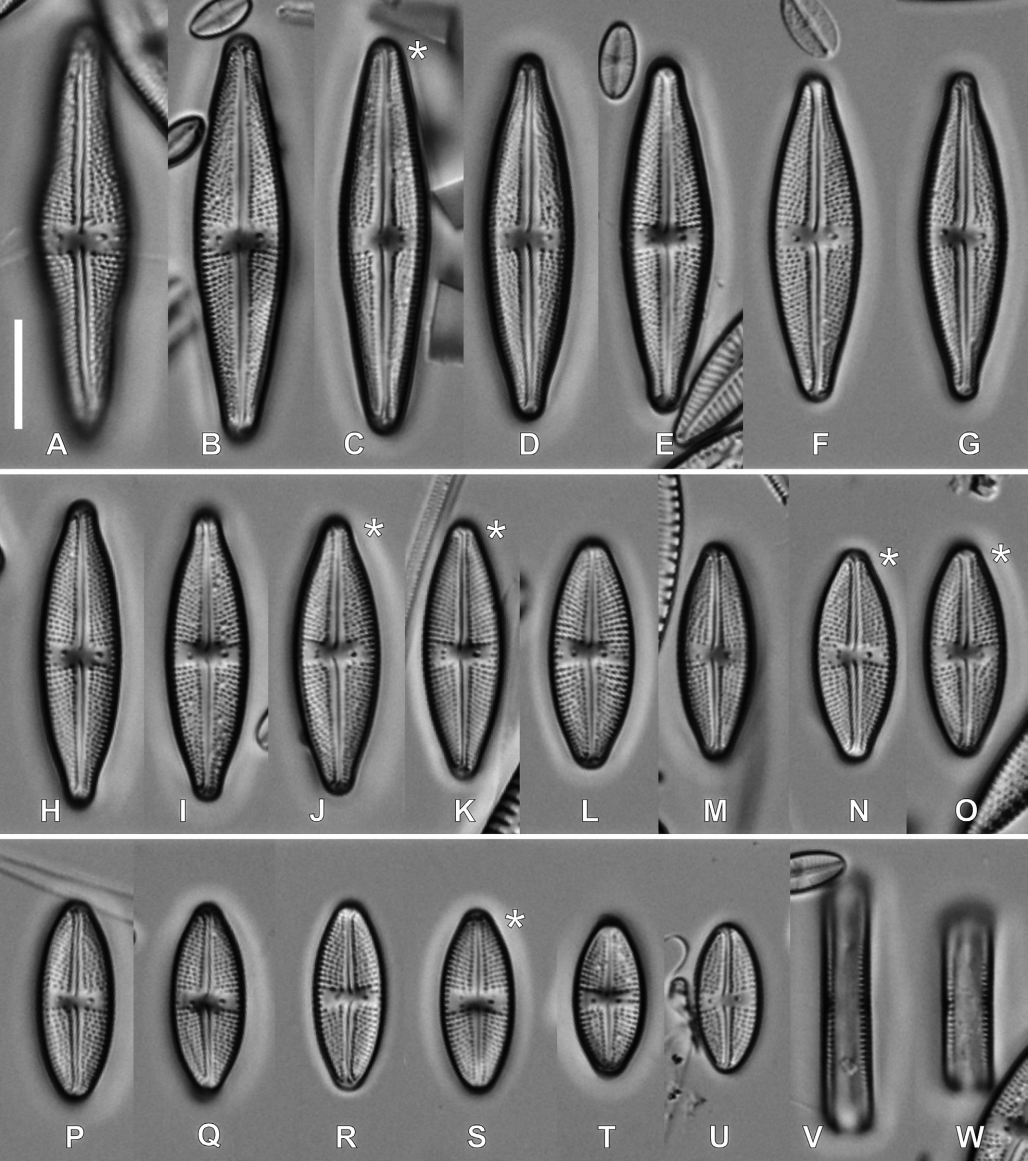
LM microphotographs of *Luticolapoliporea* sp. nov. in size diminution series. * – indicates valves with triple isolated pores. **A** initial valve, **V, W** frustule in girdle view. Scale bar: 10 µm.

Figs 3A–W , 8A–G , 9A–G

#### Description.

**LM observations (Fig. 3A–W).** Frustules rectangular in girdle view, lanceolate or linear-lanceolate with rostrate apices. Range of valve dimensions (n = 100): 12.5–39.0 μm long, 5.3–9.0 μm wide and 20–24 striae in 10 µm. Striae clearly punctate. Central area slightly bow-tie-shaped, axial area linear and narrow. Isolated pores clearly visible in central area. Usually two isolated pores are present, in about 2% of the type population, a third isolated pore occurs (Fig. 3C, J, K, T, A, C).

**SEM observations (Figs 8A–G, 9A–G). *External view***: Striae composed of 4–5 slightly elongated areolae, almost equal in size (Fig. 8A–G). Single row of elongated areolae, interrupted at valve apices, present on the valve mantle (Fig. 8G, 9G). Central area bordered by three areolae (Fig. 8A–F), ghost areolae commonly present in central area (Fig. 8A–F). Raphe filiform located on distinct raphe sternum (Fig. 8A–G). Distal raphe endings hooked and continued on to valve mantle (Fig. 8G), proximal raphe endings weakly deflected and finishing slightly pore-like enlarged (Fig. 8A–E). External openings of isolated pores elongated located in mid-way between valve margin and valve centre. Isolated pore opening on side where raphe endings are deflected commonly are smaller and shifted from centre part into striae (Fig. 8A–E). Edge of the valve mantle notched approximately half-way between centre and pole in each quadrant of the valve (Fig. 9C).

***Internal view***: Areolae occluded by hymenes forming continuous strip (Fig. 9A–D). Raphe slits straight (Fig. 9A–E). Proximal raphe endings simple and straight (Fig. 9A–C, E, F), distal raphe endings forming small helictoglossa (Fig. 9C, D). Isolated pore openings with large circular structure (Fig. 9A–C, E, F), openings of additional pores commonly reduced (Fig. 9B, C, F). Longitudinal channels visible along valve (Fig. 9A–F).

#### Type.

Indonesia, Malang, East Java, unnamed spring, 7°50'26.2"S, 112°31'43.0"E, coll. 2 July 2023. Holotype slide no. SZCZ28794! and unmounted material with the same number in the Szczecin Diatom Collection (University of Szczecin, Poland), isotype slide no. 2023/81 and the unmounted material with the same number at the University of Rzeszów, Poland. The type population is illustrated in Figs 3A–W, 8A–G, 9A–G.

#### Etymology.

Name refers to the unusual feature of bearing multiple isolated pores.

#### Distribution.

So far, species observed only in the type locality.

#### Ecology and associated diatom flora.

The species was most abundant in a sample of moss from collected from spring – where it reached 30% of the total share in diatom assemblage; together with the described species, also occurred: *Diadesmisconfervacea* Kützing, *Mayamaea* sp., various *Nitzschia* spp., Naviculacf.germainii Wallace and *Sellaphoranigrii* (De Notaris) Wetzel & Ector. The species was also observed in samples of other habitats from the same spring (sediments, epilithon), but in lower numbers.

## ﻿Discussion

Five *Luticola* observed and described in the study show specific features that separate them from all other described *Luticola* species so far. Tables 1–5 present morphological comparisons amongst all of the new species and the most similar taxa worldwide.

**Figure 4.**
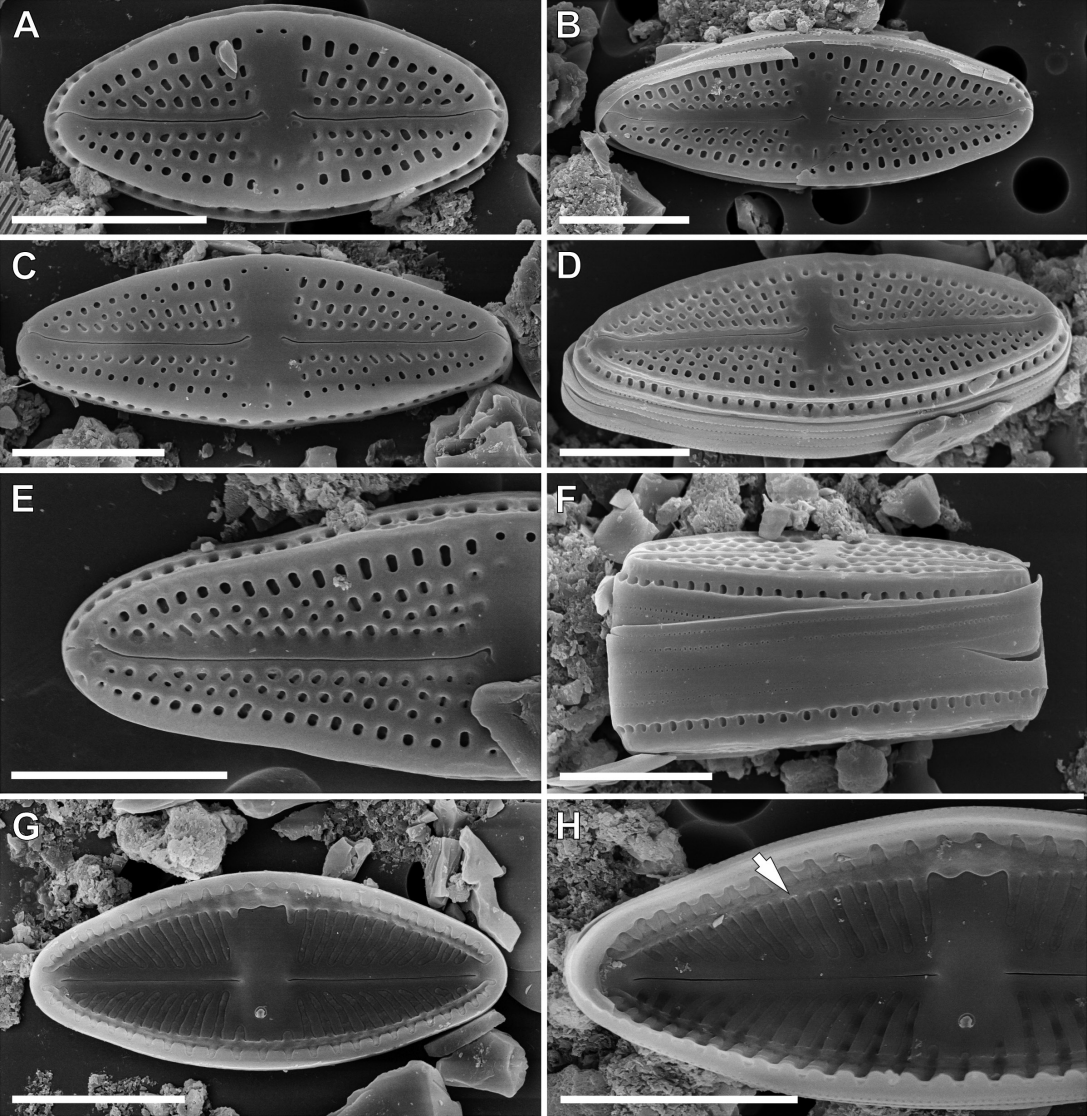
SEM microphotographs of *Luticolainsularis* sp. nov. external (**A–F**) and internal (**G, H**) views. Arrow indicate a longitudinal channel. Scale bars: 5 µm.

*Luticolainsularis* sp. nov. is morphologically most similar to *L.aequatorialis* (Heiden) Lange-Bertalot & T.Ohtsuka and *L.simplex* Metzeltin, Lange-Bertalot & García-Rodríguez, since they share similar valve outlines, areolae densities and distal raphe ending morphologies (Metzeltin et al. 2005; Levkov et al. 2013). However, *L.insularis* sp. nov. can be easily distinguished from the other two taxa by a lower striae density and narrower valves (Table 1). Moreover, *L.insularis* sp. nov. shows well-developed silica ridges along the valve face/mantle junction (Fig. 4D–F), which are absent in both *L.aequatorialis* and *L.simplex* (Metzeltin et al. 2005; Levkov et al. 2013). Additionally, the European *L.pseudoimbricata* Levkov, Metzeltin & Pavlov shows some degree of similarity and the dimensions of the two taxa overlap. However, *L.insularis* has different proximal and distal raphe endings. In *L.insularis*, the external proximal raphe endings are deflected with small, shallow grooves (Fig. 4A–E), while in *L.pseudoimbricata*, proximal raphe endings are short, curved and slightly expanded (Levkov et al. 2013; pl 16, figs 1–3, 5). Additionally, *L.insularis* has a lower striae density (16–20/10 µm vs. 20–24/10 µm) (see Table 1).

**Table 1.** Comparison of valve features between *Luticolainsularis* sp. nov. and similar species. *- indicates data obtained from photography in original description.

	*L.insularis* sp. nov.	*L.aequatorialis*	*L.pseudoimbricata*	*L.simplex*
length [µm]	8.5–24.0	15.5–32.5	9.0–22.0	9.5–26.0
width [µm]	4.6–6.7	5.2–8.6	5.0–7.0	5.0–8.0
striae [in 10 µm]	16–20	20–22	20–24	19–21
areolae [per stria]	3–4	3–4	2–4*	3–5
proximal raphe endings	deflected with small, shallow grooves	clearly deflected	short, curved and slightly expanded	doubly curved
distal raphe endings	weakly hooked	strongly hooked	short, bent	hooked
source	this study	Levkov et al. (2013)	Levkov et al. (2013)	Metzeltin et al. (2005); Levkov et al. (2013)

*Luticolabandanensis* sp. nov. resembles two European species: *L.frequentissima* Levkov, Metzeltin & Pavlov, *L.pitranensis* Levkov, Metzeltin & Pavlov and also with *L.rapanuiensis* M.Rybak, Peszek, A.Witkowski & Lange-Bertalot, which was recently described from Easter Island (Table 2). *Luticolafrequentissima* seems the most morphologically similar to *L.bandanensis* sp. nov. The two taxa can be separated, based on the valve width (4.5–6.5 µm in *L.bandanensis* sp. nov. vs. 6.5–9.0 µm in *L.frequentissima*; Levkov et al. (2013)). Additionally, *L.bandanensis* sp. nov. has symmetrical valves, while the valves of *L.frequentissima* are commonly more bulged on the side opposite the isolated pore (Levkov et al. 2013; Noga et al. 2017). Both species show irregular grooves on both distal and proximal raphe endings. In *L.bandanensis* sp. nov., these grooves are present on the side opposite to the isolated pore, while in *L.frequentissima*, the grooves extend to the isolated pore side (Levkov et al. 2013: pl. 9, figs 1–4, Noga et al. 2017: fig. 3). *Luticolapitranensis* can be distinguished from *L.bandanensis* sp. nov., based on its lower striae density (18–21/10 µm vs. 21–23/10 µm), more cuneate valve apices and, as in *L.frequentissima*, the grooves on the proximal raphe endings that extend to the isolate pore side (Levkov et al. 2013: pl. 9, figs 1–5). *Luticolarapanuiensis* can be separated from *L.bandanensis* sp. nov. by its lower striae density (16–19/10 µm vs. 21–23/10 µm), central area that is more rectangular in shape (Peszek et al. 2021: fig. 3A–P) and the presence of irregular silica ridges on the valve face/mantle junction (Peszek et al. 2021: fig. 3U, X–Z) (absent in *L.bandanensis* sp. nov.).

**Figure 5.**
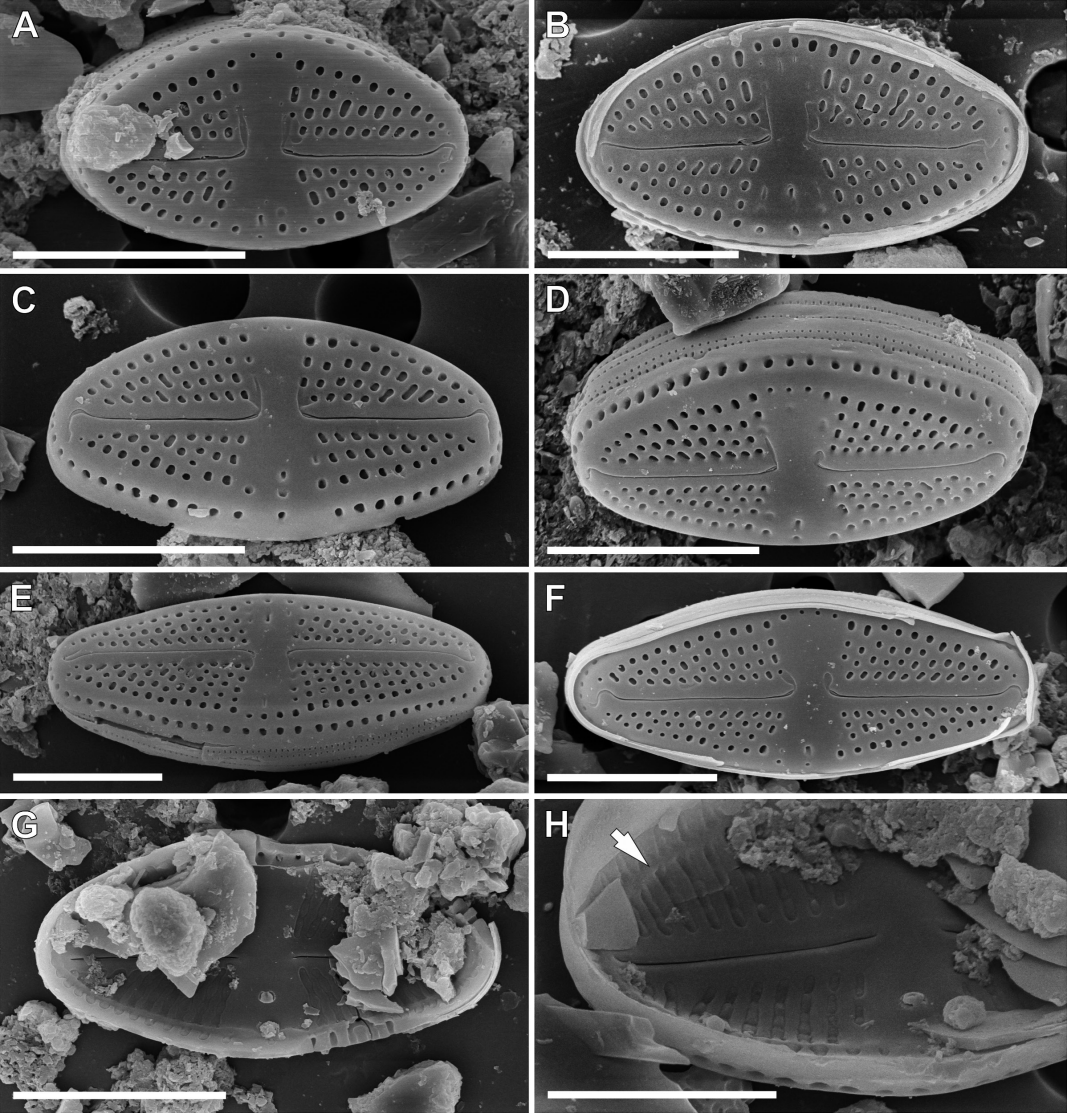
SEM microphotographs of *Luticolabandanensis* sp. nov. external (**A–F**) and internal (**G, H**) views. Arrow indicate a longitudinal channel. Scale bars: 5 µm (**A–G**); 3 µm (**H**).

**Table 2.** Comparison of valve features between *Luticolabandanensis* sp. nov. and similar species. *- indicates data obtained from photography in original description.

	*L.bandanensis* sp. nov.	*L.frequentissima*	*L.pitranensis*	*L.rapanuiensis*
length [µm]	6.8–21.5	12.0–27.0	13.5–27.0	5.2–15.9
width [µm]	4.5–6.5	6.5–9.0	5.0–7.0	4.3–7.6
striae [in 10 µm]	21–23	20–24	18–21	16–19
areolae [per stria]	3–5	4–6	4–5	2–3(4)
proximal raphe endings	deflected with irregular shallow grooves on side opposite to isolated pore	short with irregular shallow grooves on isolated pore side	hooked with irregular shallow grooves* on isolated pore side	curved towards stigma with irregular shallow grooves
distal raphe endings	hooked with irregular shallow grooves on isolated pore side	curved with irregular shallow grooves on isolated pore side	hooked	curved to tightly hooked to isolated pore side
source	this study	Levkov et al. (2013); Noga et al. (2017)	Levkov et al. (2013)	Peszek et al. (2021)

*Luticolaelliptica* sp. nov. shows some degree of similarity to *L.bryophila* M.Rybak, Czarnota & Noga, *Luticolasparsipunctata* Levkov, Metzeltin & Pavlov, *L.tenuis* Levkov, Metzeltin & Pavlov and an unidentified species from the Île Saint-Paul (Chattová et al. 2017: p. 10, figs 72–82). The European *L.sparsipunctata* can be distinguished from *L.elliptica* sp. nov., based on its wider valves with lower striae and areolae density (see Table 3) and a more lanceolate valve shape, especially in larger specimens (Levkov et al. 2013: pl. 32). Both *L.tenuis* and *L.bryophila* have a lower striae density than *L.elliptica* sp. nov. The distal endings in *L.bryophila* are short and deflected to the side opposite the isolated pore (Rybak et al. 2023: fig. 2Y–AA), while the raphe endings in *L.elliptica* sp. nov. are weakly hooked and continue on to the valve mantle (Fig. 5A–G). Finally, *Luticolaelliptica* sp. nov. is the only species with a strongly asymmetrical central area. The side bearing the isolated pore is almost two times wider than the other side. This very rare feature was only observed in a single unnamed species from Île Saint-Paul Island in the south Indian Ocean (Chattová et al. 2017). Both taxa overlap in valve shape, basic dimensions and raphe ending morphology (Table 3). However, because of the lack of SEM documentation for *Luticola* sp., it is impossible to determine definitively whether it is an isolated population of the same taxon or a similar, but different species.

**Table 3.** Comparison of valve features between *Luticolaeliptica* sp. nov. and similar species. *- indicates data obtained from photography in original description.

	*L.eliptica* sp. nov.	*L.sparsipunctata*	*L.tenuis*	*L.bryophila*	*Luticola* sp.
length [µm]	9.0–20.0	11.0–28.0	11.0–30.0	10.0–25.0	10.7–20.5
width [µm]	4.3–5.5	5.0–7.0	4.0–6.0	4–6	4.5–6.5
striae [in 10 µm]	20–24	17–20	18–20	18–20	20–24
areolae [per stria]	2–3	1–2	2–4	2–3	3–4*
proximal raphe endings	deflected away from the isolated pore	deflected away from the isolated pore	distinctly deflected away from the isolated pore	deflected away from the isolated pore	deflected away from the isolated pore
distal raphe endings	weakly hooked	short and weakly deflected or hooked	weakly hooked	deflected	deflected
source	this study	Levkov et al. (2013)	Levkov et al. (2013)	Rybak et al. (2023)	Chattová et al. (2017)

*Luticolamalukuana* sp. nov. most closely resembles *L.dismutica* (Hustedt) D.G.Mann and *L.areolata* V.Lokhande, Lowe, Kociolek & B.Karthick. The basic feature separating it from both species is the notably higher density of striae (Table 4). *L.malukuana* sp. nov. can also be distinguished from *L.dismutica* based on valve outline, which is more undulate in the latter species (see Levkov et al. 2013, pl. 151, figs 15–33, pl. 155, figs 26–35). *Luticolamalukuana* sp. nov. and *L.areolata* share a similar, slightly undulating valve outline; however, *L.areolata* has more elongated, narrower valve apices (Lokhande et al. 2020: figs 52–61). Additionally, these taxa differ in striae morphology. Striae of *L.areolata* are composed of fewer areolae (2–3 vs. 3–4) with a clearly reduced external opening (Lokhande et al. 2020: fig. 76). *Luticolaareolata* also commonly has ghost areolae in the central area (Lokhande et al. 2020: 52–61) and these are absent or rare in *L.malukuana* sp. nov. (Fig. 7A–D).

**Figure 6.**
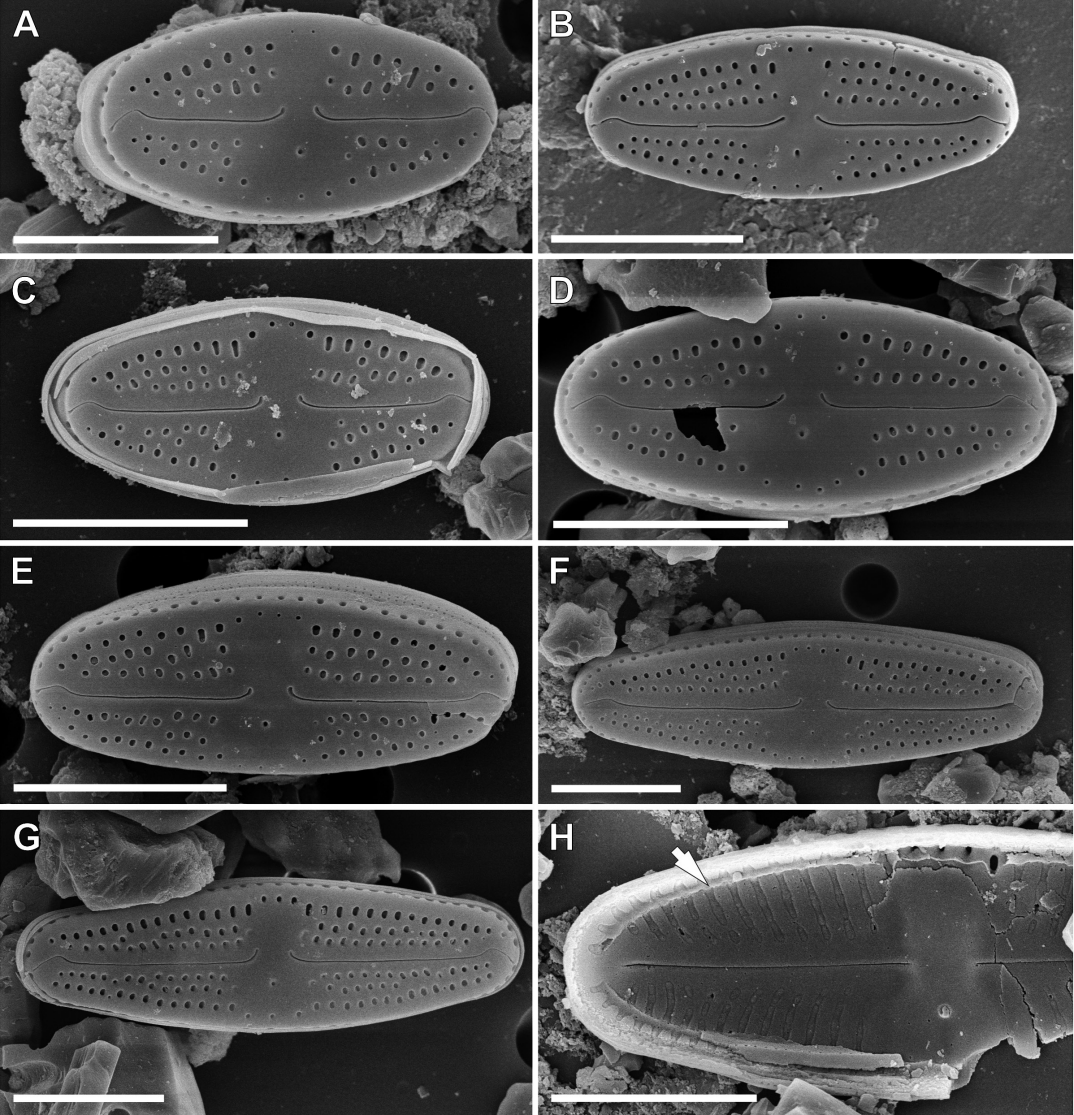
SEM microphotographs of *Luticolaeliptica* sp. nov. external (**A–G**) and internal (**H**) views. Arrow indicate a longitudinal channel. Scale bars: 4 µm (**A, C–G**); 5 µm (**B, H**).

**Figure 7.**
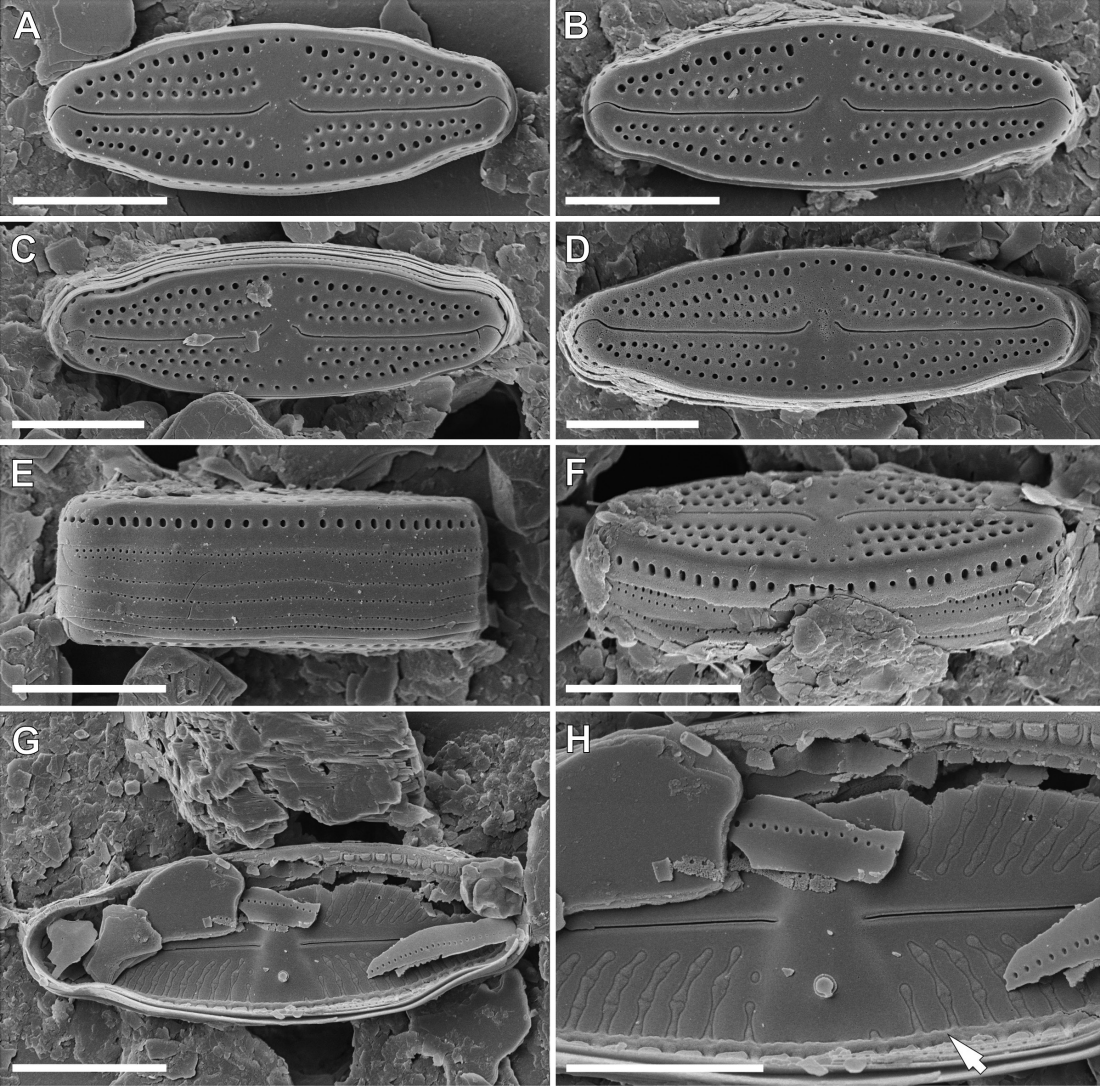
SEM microphotographs of *Luticolamalukuana* sp. nov. external (**A–F**) and internal (**G, H**) views. Arrow indicate a longitudinal channel. Scale bars: 5 µm (**A–G**); 3 µm (**H**).

**Figure 8.**
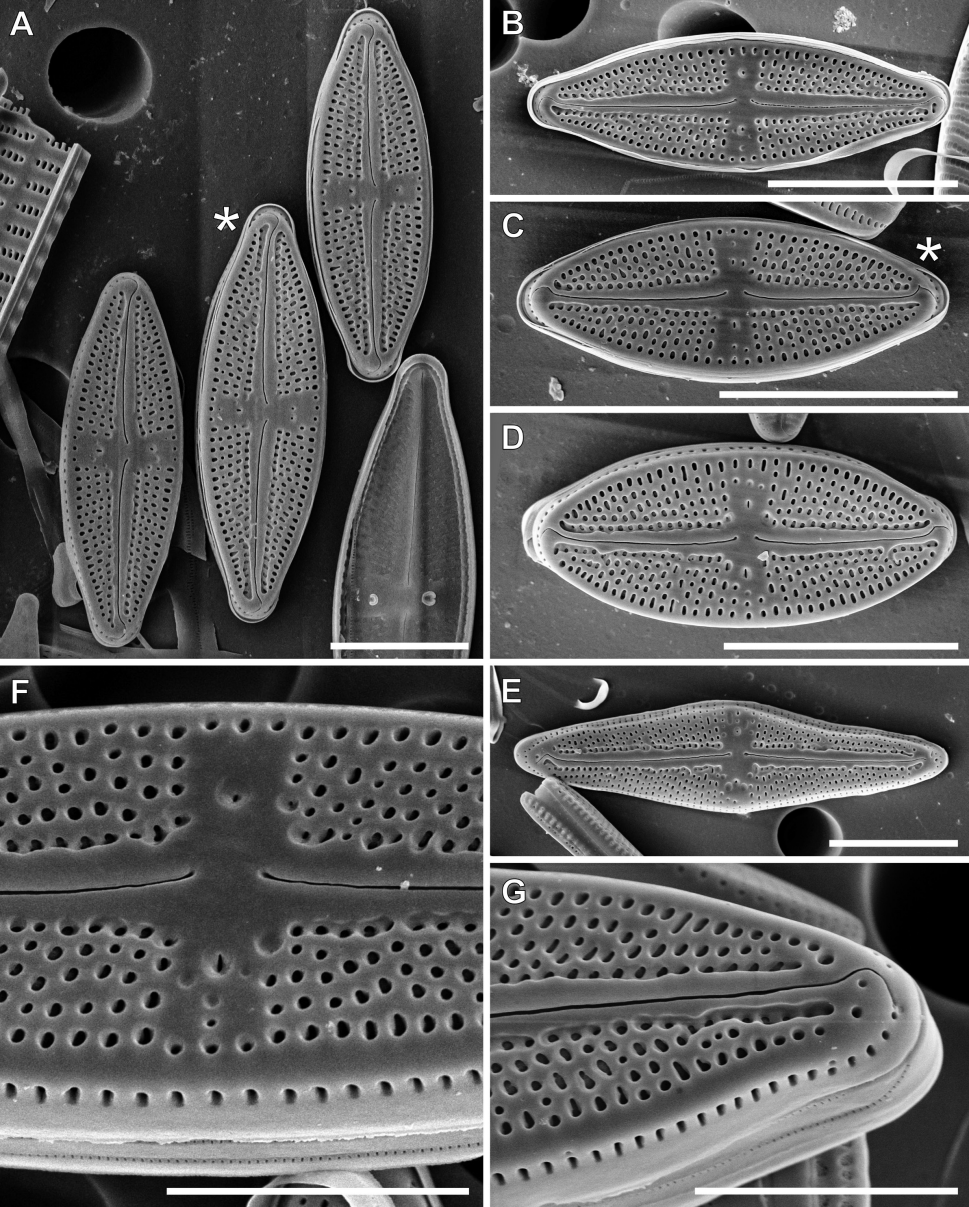
SEM microphotographs of *Luticolapoliporea* sp. nov. in external views. * – indicates valves with triple isolated pores. Scale bars: 10 µm (**A–E**); 5 µm (**F, G**).

**Figure 9.**
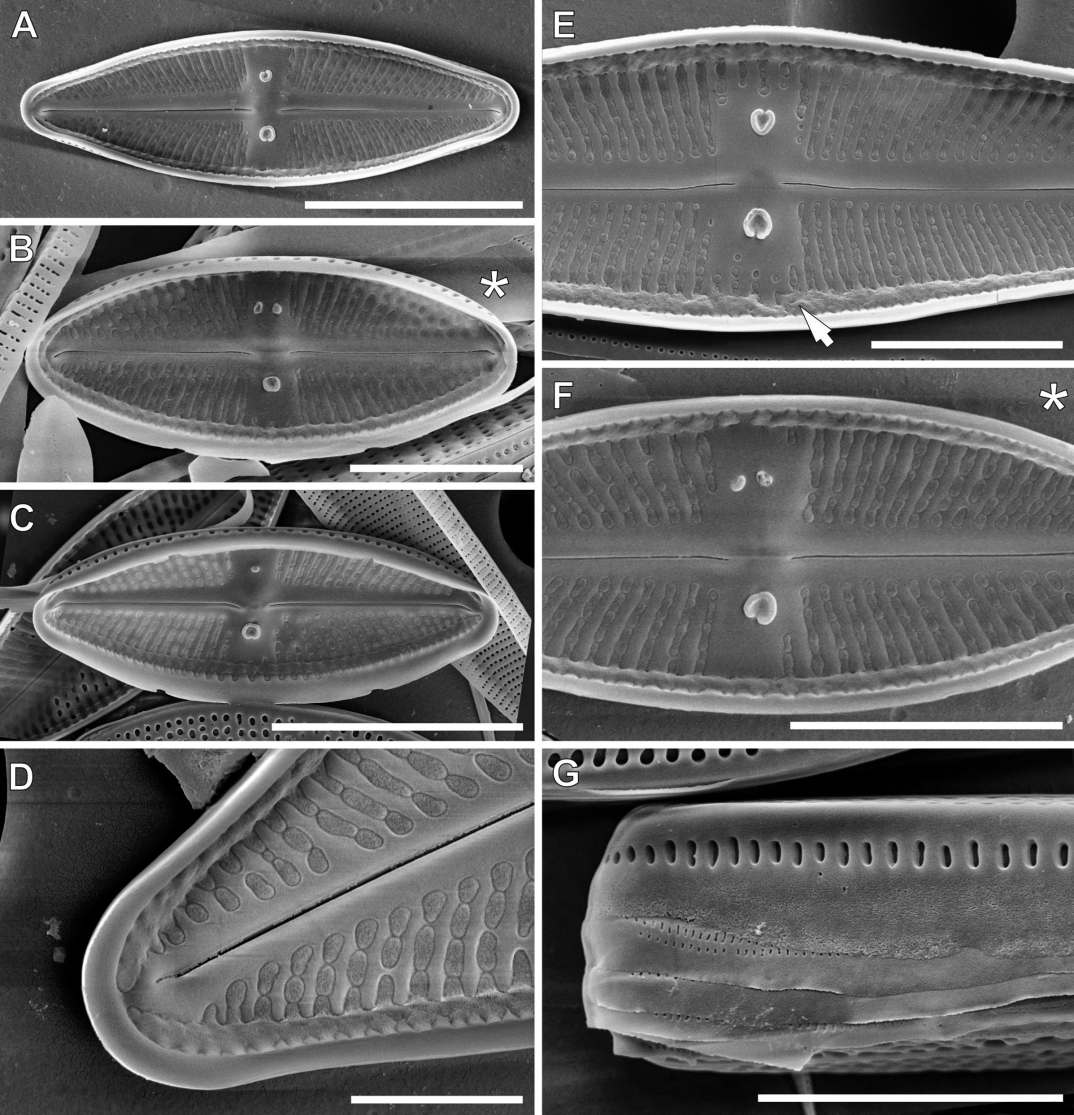
SEM microphotographs of *Luticolapoliporea* sp. nov. in internal views (**A–F**) and external details of valve mantle and girdle band (**G**). * – indicates valves with triple isolated pores, arrow indicates a longitudinal channel. Scale bars: 10 µm (**A, C**); 5 µm (**B, E–G**); 2 µm (**D**).

**Table 4.** Comparison of valve features between *Luticolamalukuana* sp. nov. and similar species. *- indicates data obtained from photography in original description.

	*L.malukuana* sp. nov.	*L.dismutica*	*L.areolata*
length [µm]	12.0–28.0	18.0–40.0	23.0–37.0
width [µm]	5.5–7.0	6.0–9.5	5.5–8.5
striae [in 10 µm]	18–21	16–18	13–16
areolae [per stria]	3–4	3–4	2–3*
proximal raphe endings	deflected away from the isolated pore	weakly curved	deflected away from the isolated pore
distal raphe endings	hooked	hooked	deflected
source	this study	Levkov et al. (2013)	Lokhande et al. (2020)

*Luticolapoliporea* sp. nov. most closely resembles taxa from the group of *Luticolagoeppertiana* (Bleisch) D.G.Mann ex Rarick, S.Wu, S.S.Lee & Edlund. Amongst them, *Luticolatujii* Levkov, Metzeltin & Pavlov and *Luticolaburmensis* Metzeltin & Levkov (Levkov et al. 2013) are most similar to the new taxon (Table 5). *Luticolapoliporea* sp. nov., together with the taxa it most closely resembles, are characterised by having the raphe located on a clearly visible raphe sternum (Levkov et al. 2013: pl. 63, figs 1–7, Pl. 69, figs 1–5). However, due to the presence of multiple isolated pores in the central area, *Luticolapoliporea* sp. nov. is distinguished from both mentioned taxa. Additionally, *Luticolatuji* in contrast to newly-described species, has narrower and usually shorter valves with denser striae (Levkov et al. 2013). *Luticolaburmensis* can be distinguished from *L.poliporea* sp. nov. by less rostrate apices and mostly wider valves (Levkov et al. 2013). Moreover, multiple isolated pores make this species unique amongst the entire genus. So far, all taxa described in the genus *Luticola* have a single isolated pore on each valve (Round et al 1990).

**Table 5.** Comparison of valve features between *Luticolapoliporea* sp. nov. and similar species.

	*L.poliporea* sp. nov	*L.tuji*	*L.burmensis*
length [µm]	12.5–39.0	14.0–23.0	20.0–36.0
width [µm]	5.3–9.0	5.0–6.5	7.0–10.5
striae [in 10 µm]	20–24	22–28	19–22
areolae [per stria]	4–5	3–5	4–5
proximal raphe endings	deflected to the side opposite to isolated pore	deflected to the side opposite to isolated pore	deflected to the side opposite to isolated pore
distal raphe endings	hooked	hooked	hooked
source	this study	Levkov et al. (2013)	Levkov et al. (2013)

Species of the genus *Luticola* described in recent years, especially those representing the *L.goeppertiana* group (Simonato et al. 2020; Yang et al. 2022) clearly show that the morphological boundaries of the genus are much wider than previously documented (Round et al. 1990; Levkov et al. 2013). The original description of the genus by Round et al. (1990) indicated several distinguishing features of the genus *Luticola*, such as: mainly solitary life habit, single plastid with two lobes, single central pyrenoid, mantle margin notched halfway between the apices and valve middle, uniseriate striae, single row of areolae on valve mantle, poroids on valve face and mantle covered by hymenes, presence of a longitudinal canal, narrow raphe-sternum and short stauros, presence of single isolated pore (called stigma) with lipped external opening, deflected raphe endings and open girdle bands with 1 or 2 row of poroids. Since the genus was described, several species with multiple rows of areolae on the mantle have been described or transferred to the genus (Mayama and Kawashima 1998; Pavlov et al. 2009; Wetzel et al. 2010; Da Silva Lehmkuhl et al. 2019; Simonato et al. 2020).

Levkov et al. (2013), in their monograph of the genus, draw attention to the great heterogeneity of the genus in terms of the shape of frustules, areolae and raphe ends. They also draw attention to individual taxa that deviate from the general pattern of frustule structure due to the presence of distinct ridges or spines on the edge. Additionally, Levkov et al. (2013) noted that *L.mutica* (which is the type species of the genus), unlike the other species known at that time, does not have simple areoles, but has a recessed cribrum (Levkov et al. 2013). However, since the publication of the monograph of the genus, several additional species possessing a cribrum have been described, i.e. *Luticolaivetana* Chattova & B.Van de Vijver, *L.cribriareolata* M.Rybak, Witkowski, Risjani & Yunianta and *L.jinshaensis* L.Yang & Q.X.Wang (Chattová et al. 2017; Rybak et al. 2021b; Yang et al. 2022). Additionally, Simonato et al. (2020) shed some light on the morphological variability of the genus, pointing out the occurrence of pseudosepta in some species group, as well as the absence of longitudinal/marginal channels in others. Observations of various species show that the range of morphological variability of the genus is much broader than originally assumed by Round et al. (1990), within which taxa demonstrating certain differences from many characteristic valve features are found (i.e. taxa without longitudinal/marginal canals, having additional rows of areolae on the mantle, additional isolated pores, cribra-bearing). Despite the significant expansion of knowledge about the diversity of morphological features found within *Luticola*, observations of living cells so far show that features, such as the shape of the plastid, the number of its lobes and the presence of a single pyrenoid, seem to be constant (Denys and De Smet 1996; Poulíčková 2008; Bagmet et al. 2023).

A significant number of new diatom taxa have been described in the last few years from both terrestrial and freshwater environments in Southeast Asia. The discovery of five new species of *Luticola* in just three samples presented herein, shows that the poorly-explored terrestrial environments hide interesting and yet undescribed species.

## Supplementary Material

XML Treatment for Luticola insularis

XML Treatment for Luticola bandanensis

XML Treatment for Luticola elliptica

XML Treatment for Luticola malukuana

XML Treatment for Luticola poliporea

## ﻿Additional information

### Author ORCIDs

Mateusz Rybak https://orcid.org/0000-0001-8998-9537

Łukasz Peszek https://orcid.org/0000-0002-9132-2210

Oktiyas Muzaky Luthfi https://orcid.org/0000-0002-9550-9381

Sulastri Arsad https://orcid.org/0000-0002-7322-7834

John Patrick Kociolek https://orcid.org/0000-0001-9824-7164

Andrzej Witkowski http://orcid.org/0000-0003-1714-218X

### Data availability

All of the data that support the findings of this study are available in the main text.

## References

[B1] Bagmet VB Abdullin SR Nikulin AY Nikulin VY Gontcharov AA (2023) *Luticolatenera* sp. nov. (Diadesmidaceae, Naviculales) – A new diatom from the soil of the State Nature Reserve “Bastak” (Jewish Autonomous Region, Russia). Life 13(1937): 1–13. 10.3390/life13091937 PMC10533167 37763341

[B2] Barber HG Haworth EY (1981) A guide to the morphology of the diatom frustule with a key to the British freshwater genera. Scientific Publication – Freshwater Biological Association 44: 1–112.

[B3] Bishop J Wasley J Waterman M Kohler T Van de Vijver B Robinson S Kopalová K (2021) Diatom communities differ among Antarctic moss and lichen vegetation types. Antarctic Science 33(2): 118–132. 10.1017/S0954102020000620

[B4] Chattová B (2018) Diatoms (Bacillariophyta) associated with lichens from Ulu Peninsula (James Ross Island, NE Antarctic Peninsula). Czech Polar Reports 8(2): 151–161. 10.5817/CPR2018-2-12

[B5] Chattová B Lebouvier M De Haan M Van de Vijver B (2017) The genus *Luticola* (Bacillariophyta) on Ile Amsterdam and Ile Saint-Paul (Southern Indian Ocean) with the description of two new species. European Journal of Taxonomy 387(387): 1–17. 10.5852/ejt.2017.387

[B6] Chattová B Cahová T Pinseel E Kopalová K Kohler TJ Hrbacek F Van de Vijver B Nývlt D (2022) Diversity, ecology, and community structure of the terrestrial diatom flora from Ulu Peninsula (James Ross Island, NE Antarctic Peninsula). Polar Biology 45(1): 873–894. 10.1007/s00300-022-03038-z

[B7] Da Silva Lehmkuhl AM Ludwig TAV Tremarin PI Bicudo D (2019) On *Luticola* Mann (Bacillariophyceae) in southeastern Brazil: Taxonomy, ecology and description of two new species. Phytotaxa 402(4): 165–186. 10.11646/phytotaxa.402.4.1

[B8] Denys L De Smet WH (1996) Observations on the subaerial diatom *Naviculaspinifera* Bock, and its transfer to *Luticola* Mann. Cryptogamie. Algologie 17: 77–93.

[B9] Glushchenko AM Kulikovskiy MS (2015) Species of the genus *Luticola* in waterbodies of Laos and Vietnam. Botanicheskii Zhurnal 100(8): 799–804.

[B10] Glushchenko AM Kulikovskiy MS Kociolek JP (2017) New and interesting species from the genus *Luticola* (Bacillariophyceae) in waterbodies of Southeastern Asia. Nova Hedwigia. Beiheft 146: 157–173. 10.1127/1438-9134/2017/157

[B11] Glushchenko AM Kuznetsova IV Kulikovskiy MS (2021) The Diatoms of Southeast Asia. Yaroslavl, Filigran, 320 pp.

[B12] Glushchenko A Kezlya E Maltsev Y Genkal S Kociolek JP Kulikovskyi M (2022) Description of the Soil Diatom *Sellaphoraterrestris* sp. nov. (Bacillariophyceae, Sellaphoraceae) from Vietnam, with Remarks on the Phylogeny and Taxonomy of *Sellaphora* and Systematic Position of *Microcostatus*. Plants 11(16): e2148. 10.3390/plants11162148 PMC9415725 36015452

[B13] Grunow A (1865) Über die von Herrn Gerstenberger in Rabenhorst›s Decaden ausgegeben Süsswasser Diatomaceen und Desmidiaceen von der Insel Banka, nebst Untersuchungen über die Gattungen *Ceratoneis* und *Frustulia*. In: Rabenhorst L (Ed.) Beiträge zur Näheren Kenntniss und Verbreitung der Algen. Verlag von Eduard Kummer, Leipzig, Heft II, 16 pp.

[B14] Guiry MD Guiry GM (2023) AlgaeBase. World-wide electronic publication, National University of Ireland, Galway. https://www.algaebase.org [Accessed 6 February 2023]

[B15] Kale A Levkov Z Karthick B (2017) Typification of two species of *Luticola* (Bacillariophyta) from aerophilic habitats of the Western Ghats, India. Phytotaxa 298(1): 29–42. 10.11646/phytotaxa.298.1.3

[B16] Kezlya E Glushchenko A Maltsev Y Gusev E Genkal S Kuznetsov A Kociolek JP Kulikovskyi M (2020a) *Placoneiscattiensis* sp. nov.—a new, diatom (Bacillariophyceae: Cymbellales) soil species from Cát Tiên National Park (Vietnam). Phytotaxa 460(4): 237–248. 10.11646/phytotaxa.460.4.1

[B17] Kezlya E Glushchenko A Kociolek JP Maltsev Y Martynenko N Genkal S Kulikovskyi M (2020b) *Mayamaeavietnamica* sp. nov.: A new, terrestrial diatom (Bacillariophyceae) species from Vietnam. Algae – Korean Phycological Society 35(4): 325–335. 10.4490/algae.2020.35.11.23

[B18] Kezlya E Glushchenko A Kociolek JP Maltsev Y Genkal S Kulikovskyi M (2022a) A new species of *Placoneis* Mereschkowsky (Bacillariophyceae: Cymbellales) from wet soils in southern Vietnam. Cryptogamie. Algologie 43(11): 177–188. 10.5252/cryptogamie-algologie2022v43a11

[B19] Kezlya E Maltsev Y Genkal S Krivova Z Kulikovskyi M (2022b) Phylogeny and fatty acid profiles of new *Pinnularia* (Bacillariophyta) species from Soils of Vietnam. Cells 11(15): e2446. 10.3390/cells11152446 PMC9368540 35954290

[B20] Kolkwitz R Krieger W (1936) Zur Ökologie der Pflanzenwelt, insbesondere der Algen, des Vulkans Pangerango in West-Java. Berichte der Deutschen Botanischen Gesellschaft 54(2): 65–91. 10.1111/j.1438-8677.1936.tb01946.x

[B21] Levkov Z Metzeltin D Pavlov A (2013) *Luticola* and *Luticolopsis*. Diatoms of the European inland waters and comparable habitats. Diatoms of Europe 7: 1–698.

[B22] Liu B Williams DM Blanco S Jiang X (2017) Two new species of *Luticola* (Bacillariophyta) from the Wuling Mountains Area, China. Nova Hedwigia. Beiheft 146: 197–208. 10.1127/1438-9134/2017/197

[B23] Lokhande V Radhakrishnan C Kociolek JP Lowe R Karthic B (2020) The diatom genus *Luticola* D.G.Mann (Bacillariophyceae) in the Western Ghats of India and its biogeography. European Journal of Phycology 56(2): 142–158. 10.1080/09670262.2020.1783460

[B24] Mayama S Kawashima A (1998) New combinations for some taxa of *Navicula* and *Stauroneis*, and an avowed substitute for a taxon of *Eunotia*. Diatom. The Japanese Journal of Diatomology 14: 69–71. 10.11464/diatom1985.14.0_69

[B25] Metzeltin D Lange-Bertalot H García-Rodriguez F (2005) Diatoms of Uruguay. Compared with other taxa from South America and elsewhere. Iconographia Diatomologica 15: 1–736.

[B26] Noga T Stanek-Tarkowska J Kochman-Kędziora N Rybak M Peszek Ł Poradowska A (2017) *Luticolafrequentissima* Levkov, Metzeltin & Pavlov – morphological and ecological characteristics of a population from Southern Poland. Oceanological and Hydrobiological Studies 46(2): 237–243. 10.1515/ohs-2017-0024

[B27] Pavlov A Nakov T Levkov Z Furey P Lowe R Ector L (2009) *Luticolagrupcei* (Bacillariophyceae) – a new freshwater diatom from Mountain Baba (Macedonia) and Great Smoky Mountains National Park (U.S.A.): Comparison with the type material of *L.goeppertiana* (Bleisch) D.G.Mann. Nova Hedwigia 89(1/2): 147–164. 10.1127/0029-5035/2009/0089-0147

[B28] Peszek Ł Rybak M Lange-Bertalot H Kociolek JP Witkowski A (2021) Three new *Luticola* D.G.Mann (Bacillariophyta) species from Rapa Nui (Easter Island) found in terrestrial diatom assemblages dominated by widely distributed taxa. PeerJ 9: e11142. 10.7717/peerj.11142 PMC8029705 33868817

[B29] Poulíčková A (2008) Morphology, cytology and sexual reproduction in the aerophytic cave diatom *Luticoladismutica* (Bacillariophyceae). Preslia 80(1): 87–99.

[B30] Radhakrishnan C Yogeshwaran M Karthick B (2022) Hanging in the air: Tree moss diatoms from Indo-Burma biodiversity hot spot of India. Aerobiologia 38(1): 557–566. 10.1007/s10453-022-09766-3

[B31] Round FE Crawford RM Mann DG (1990) The Diatoms. Biology & Morphology of the Genera. Cambridge University Press, Cambridge, 747 pp.

[B32] Rybak M Solak CN Noga T Glushchenko A Williams DM Kulikovskyi M (2020) *Nupelabrevistriata* sp. nov. – a new, terrestrial diatom species from Southeast Asia. Diatom Research 34(4): 1–8. 10.1080/0269249X.2019.1698467

[B33] Rybak M Kochman-Kędziora N Peszek Ł (2021a) Description of four new terrestrial diatom species from *Luticola* and *Microcostatus* genera from South Africa. PhytoKeys 182: 1–26. 10.3897/phytokeys.181.65326 34616208 PMC8455506

[B34] Rybak M Witkowski A Peszek Ł Kociolek JP Risjani T Nguyen HD Zhang J Yunianta Nguyen VD Gastineau R Duong TT Rosa P Meleder V (2021b) Marine and brackish *Luticola* D.G.Mann (Bacillariophyta) species from the Java Sea and South China Sea coasts with the description of three new species. PhytoKeys 183: 115–142. 10.3897/phytokeys.183.71049 34754265 PMC8556211

[B35] Rybak M Glushchenko A Witkowski A Lange-Bertalot H Kulikovskyi M (2022a) Diversity of the genus *Orthoseira* Thwaites (Bacillariophyceae) from Southeast Asia and Rapa Nui Island with descriptions of four new taxa. Diatom Research 37(1): 1–16. 10.1080/0269249X.2022.2043448

[B36] Rybak M Kochman-Kędziora N Luthfi OM (2022b) Four novel species from the genus *Hantzschia* Grunow (Bacillariophyta: Bacillariaceae) from rural areas of Southeast Asia. Phytotaxa 567(3): 207–221. 10.11646/phytotaxa.567.3.1

[B37] Rybak M Kochman-Kędziora N Luthfi OM (2022c) A new diatom (Bacillariophyta) species from Indonesian urban areas, description of *Microcostatuslabrisicus* sp. nov. Phytotaxa 555(1): 87–94. 10.11646/phytotaxa.555.1.6

[B38] Rybak M Czarnota P Noga T (2023) Study of terrestrial diatoms in corticolous assemblages from deciduous trees in Central Europe with descriptions of two new *Luticola* D.G.Mann taxa. PhytoKeys 7(221): 1–40. 10.3897/phytokeys.221.95248 PMC10209713 37250350

[B39] Simonato J Kociolek JP Sala SE Díaz YP Núñez-Avellaneda M (2020) Three new *Luticola* species from the Andean-Amazonian transition in Colombia: Taxonomy, morphology and preliminary considerations of biogeography of the genus. Diatom Research 35(4): 377–393. 10.1080/0269249X.2020.1813205

[B40] Wetzel CE Van de Vijver B Ector L (2010) *Luticoladenisae* sp. nov. A new epizoic diatom from the Rio Negro (Amazon hydrographic basin). Vie et Milieu 60(3): 177–184.

[B41] Wu SC Bergey EA (2017) Diatoms on the carapace of common snapping turtles: *Luticola* spp. dominate despite spatial variation in assemblages. PLoS ONE 12(2): e0171910. 10.1371/journal.pone.0171910 PMC5305193 28192469

[B42] Yang L Yu P Wang Q Kociolek JP You Q (2022) *Luticolajinshaensis* sp. nov. (Bacillariophyta), a new freshwater species from Jinsha River, China. Fottea 22(1): 152–161. 10.5507/fot.2021.021

